# Nurses’ perception and compliance with personal protective equipment and hand hygiene during the third wave of COVID-19 pandemic

**DOI:** 10.1186/s42506-022-00109-1

**Published:** 2022-08-18

**Authors:** Noha Elshaer, Hesham Agage

**Affiliations:** 1 Industrial Medicine and Occupational Health, Community Medicine Department, Faculty of Medicine, Alexandria University, Alexandria, Egypt; 2 Medical student in the last grade, Faculty of Medicine, Alexandria University, Alexandria, Egypt

**Keywords:** COVID-19, Perception, Compliance, Personal protective equipment, Healthcare workers

## Abstract

**Background:**

Healthcare workers' (HCWs) compliance with infection prevention and control (IPC) measures during the COVID-19 pandemic is crucial to reducing the spread of infection to their colleagues, families, and community. This study assessed the risk perception and compliance with personal protective equipment (PPE) usage, hand hygiene, and specific IPC measures and explored the factors associated with compliance among nurses during the third wave of the COVID-19 pandemic in Egypt.

**Methods:**

A hospital-based cross-sectional survey was conducted at the Alexandria Main University Hospital (AMUH) in Alexandria city from May to August 2021, where 354 nurses were included with a response rate of 94.9%. A structured interviewer-administered questionnaire was used for data collection. Univariate and multivariate logistic regression analyses were conducted.

**Results:**

The overall compliance with PPE usage, hand hygiene, and IPC measures was 81.9%. The mean risk perception score was 40.9 ± 3.3. More than 95% of nurses were aware of the high risk of COVID-19 infection at their workplace, the serious consequences of the disease, and the risk that can be minimized by using PPE, whereas a relatively low percentage of nurses believed that the risk of COVID-19 infection could be reduced by using a surgical mask (19.2%) or gloves (50.5%). Good compliance was independently predicted by risk perception (OR = 1.25; 95% CI = 1.13, 1.39), and knowledge about PPE usage and hand hygiene (OR = 3.53; 95%CI = 2.40, 5.19). Facilitators of compliance with the PPE usage were attending suspected or confirmed COVID-19 cases in their hospital ($\overline{x}$ = 9.82), comfort to use the PPE ($\overline{x}$ = 9.16), availability of PPE ($\overline{x}$ = 8.96), hospital policy ($\overline{x}$ = 8.74), and senior compliance ($\overline{x}$ = 6.5).

**Conclusions:**

Nurses at AMUH reported high risk perceptions. The rate of compliance with PPE usage, hand hygiene, and IPC measures was 81.9%. The personal risk perception and knowledge about the PPE usage and hand hygiene are the keys to improving compliance in a healthcare facility.

## Introduction

On March 11, 2020, the World Health Organization (WHO) declared the coronavirus disease (COVID-19) as a global pandemic [1]. As of September 20, 2021, global reports mounted to more than 225 million confirmed cases and 4.5 million deaths. On the same date, Egypt reported 296,929 confirmed cases and 16,970 deaths [2]. These figures represent underreported existing cases due to insufficient resources that preclude the investigation of a larger number of individuals [3].

Healthcare workers (HCWs) are at higher risk for COVID-19 infection than the general population [4]. In healthcare settings, the most common mode of transmission of severe acute respiratory syndrome coronavirus 2 (SARS-CoV-2) is the contact of the mucosa with infectious respiratory droplets or fomites [4, 5]. Health teams at healthcare facilities located in areas with considerable community transmission are more likely to encounter patients with COVID-19 infection [6]. Although HCWs have a crucial role in reducing nosocomial transmission, they might be a source of virus transmission to their families and neighborhoods [7].

COVID-19 threatens the HCWs due to high occupational exposure, reliance on vaccination, and compliance with the recommended infection prevention and control (IPC) measures, including personal protective equipment (PPE) usage and hand hygiene [8–11]. The type of PPE used when caring for patients varies according to the setting, target personnel or patients, and type of activity [12]. For example, in the absence of aerosol-generating procedures (AGPs), the WHO recommends that HCWs providing care to patients with suspected or confirmed COVID-19 should wear a medical mask in addition to gowns, gloves, and eye protection (goggles or face shield) as a part of droplet and contact precautions, whereas in settings where AGPs are performed, HCWs should wear respirators (N95, FFP2, FFP3) in addition to eye protection, gloves, and gowns as a part of airborne and contact precautions [13].

However, a wide variation in HCWs’ understanding of the use of PPE was noted [4]. Studies conducted in low-income countries during the COVID-19 era to evaluate HCWs’ knowledge and compliance have shown diverging results [14–18]. Elsokkary et al.’s study (2021) observed that the compliance of HCWs in Egypt during the first wave of the pandemic was 46.8% [19].

Factors influencing the HCWs’ compliance with IPC measures could be HCW-related factors (such as gender, age, profession, knowledge, and perception) and organization-related factors (such as availability of PPE, IPC guidelines, training, workload, and hospital policy) [20, 21]. Within a healthcare facility, the identification of barriers and facilitators to compliance would be important to reduce infection transmission by HCWs. This study assessed the risk perception and compliance with the PPE usage, hand hygiene, and specific IPC measures for COVID-19 disease prevention and explored the factors associated with compliance among nurses at the Alexandria Main University Hospital (AMUH) during the third wave of COVID-19 pandemic in Egypt.

## Methods

### Research design and setting

A hospital-based cross-sectional survey was conducted during the third wave of COVID-19 pandemic (from May to August 2021) among nurses at AMUH which is the largest referral hospital and is located in the El-azareta district, in Alexandria city, which is the second-largest city in Egypt. AMUH provides specialized healthcare to people in Alexandria and nearby governorates.

### Participants

All registered nurses with employment duration at AMUH of at least 1 year were included. Of the 373 eligible nurses, 354 (94.9%) agreed to participate in the study in which 133 nurses were from internal medicine departments including cardiology (*n* = 51), endocrinology (*n* = 5), rheumatology (*n* = 9), diabetology (*n* = 12), geriatric medicine (*n* = 8), tropical medicine (*n* = 8), nephrology (*n* = 8), hepatology, hematology, and gastroenterology departments (*n* = 32). While 221 nurses were from surgical and emergency department, neurosurgery and intensive care unit (*n* = 34), ophthalmology (*n* = 11), ENT (*n* = 14), genitourinary (*n* = 32), head and neck (*n* = 15), surgical gastroenterology (*n* = 20), vascular (*n* = 11), cardio-thorax (*n* = 19), colorectal (*n* = 12), oncology (*n* = 13), anesthesia (*n* = 3), plastic surgery (*n* = 14), and emergency department (*n* = 23).

### Research tool

Data was collected using a pre-tested structured interviewer-administered questionnaire adopted from the previous studies, as well as the Centers for Disease Control and Prevention (CDC) and the WHO guidelines for healthcare IPC measures during the COVID-19 pandemic [7–11]. The questionnaire included 56 questions in five sections.

- Section (I) included nine questions to collect sociodemographic data (gender, age, and educational level) and occupational data (affiliation, employment duration, working hours/day, work schedule, and the number of night shifts/month).
- Section (II) included 15 questions to evaluate the compliance with the PPE usage, hand hygiene, and specific IPC measures. This section covered the frequency and extended use of various types of PPE (9 questions); compliance with the recommended steps for donning and doffing PPE while performing routine care (droplet precautions) or AGPs, and hand wash based on a detailed description of the technique (steps) (3 questions); and compliance with specific IPC measures namely not coming to work when having fever or symptoms, maintaining physical distancing (6 feet) at work even in non-patient care areas, and postponing elective time-off during the pandemic (3 questions). Each question was scored “1” for a response compliant with the recommendations, and “0” for a response not compliant and the total score was ranging from 0 (the minimum) to 15 (the maximum). The median was used as a cutoff point (7.5) to determine good compliance (median ≥ 7.5) and poor compliance (median < 7.5) [19].
- Section (III) included nine questions to evaluate nurses’ perception of the risk of COVID-19 infection using a 5-point Likert scale where strongly disagree scored “1” and strongly agree scored “5”. The total points ranged from 5 (the minimum) to 45 (the maximum). The perception assessment included their belief in the high risk of COVID-19 infection at their workplace, serious consequences of the disease, and minimizing the risk by PPE usage, hand washing, and hand sanitizer use.
- Section (IV) included eight questions to evaluate nurses’ knowledge about PPE usage and hand hygiene. The assessment covered the type of PPE used for routine care or while performing AGPs, disposable PPE and PPE that could be used for an extended period, donning and doffing PPE, indications of hand hygiene at work, and recommended steps and duration of hand wash. A score of “1” was given for a correct response and “0” for an incorrect response. The total knowledge scores ranged from 0 (the minimum) to 8 (the maximum).
- Section (V) included 15 questions to assess the availability of PPE, receiving relevant training and factors that enhance compliance with PPE usage. Each factor was assessed on a 10-point Likert scale with responses ranging from “not at all” scored “1” up to “very much” scored “10”.

### Statistical analysis

The SPSS v.20 (IBM Corp. Released 2011. IBM SPSS Statistics for Mac, Armonk, NY, USA) was used for data entry and analysis. The quantitative variables were expressed as the mean with standard deviation and qualitative variables as the frequencies and percentages. The reliability of the generated scale was tested using the Cronbach Alpha analysis [22]. Factors enhancing compliance with PPE usage (as perceived by nurses) were prioritized by the mean score for each factor.

In this study, analytic statistics included the parametric (Student’s *t* test) and non-parametric tests (chi-square test, Fisher’s exact test, and Monte-Carlo tests). A case-control approach analysis was conducted including a univariate logistic regression to find out potential sociodemographic, personal, and occupational factors associated with good compliance and calculate the odds ratio (OR) and the 95% confidence interval (95%CI).

Multivariate logistic regression analysis was conducted to model compliance as a function of the aforementioned factors to study their independent effect on compliance. The model included all participants (*n* = 354) and 11 factors namely gender, age, educational level, knowledge level, perception, history of COVID-19 disease, having a colleague or relative who had COVID-19 disease, department of affiliation, working hours/day, working schedule, and receiving training program. Collinearity was tested with variance inflation factors (VIF); a VIF value of 10 was considered large enough for problematic multicollinearity [23], and accordingly, employment duration was excluded from the model (VIF=12). The explained variance of logistic regression models was determined by Nagelkerke’s *R*^2^ and the Hosmer and Lemeshow goodness-of-fit test. All statistical analyses were judged at a level of significance of 5% (*α*=0.05).

## Results

### Sociodemographic and occupational characteristics

The majority of the 354 nurses were women (83.3%). Their mean age was 38.4 ± 10.8 years and 55.5% of them aged less than 40 years. Nearly 70% of nurses graduated from a nursing school, whereas 30% were graduated from nursing universities or institutes. The mean employment duration at AMUH was 18.3 ± 11.3 years with nearly half have been working at AMUH for ≥ 20 years. Most nurses (76.8%) worked for ≤ 8 h/day, and 45% had shift work. The majority (85%) received training on PPE usage and hand hygiene specific to the COVID-19 pandemic at AMUH (Table 1).

**Table 1:** Sociodemographic and occupational characteristics of the studied nurses at Alexandria Main University Hospital, 2021 (*n*=354)

	Frequency (No.)	Percentage (%)
Gender		
Man	34	9.6
Woman	295	83.3
Age (years)		
($\overline{x}$ ± SD: 38.4±10.8) (Min-Max: 20–59)		
< 40	196	55.4
≥ 40	158	44.6
Level of education		
Nursing school	237	66.9
Nursing Institute	117	33.1
Department		
Surgical & emergency departments	221	62.4
Internal medicine departments	133	37.6
Duration of employment (years)		
($\overline{x}$ ± SD: 18.3±11.3) (Min-Max: 1.5–40)		
< 20	185	52.3
≥ 20	169	47.7
Working hours per day (hours)		
($\overline{x}$ ± SD: 7.5±2.5) (Min-Max: 6–12)		
≤ 8	272	76.8
> 8	82	23.2
Work schedule		
Day time work	195	55.1
Shift work	159	44.9
Number of nightshifts per month (*n*=159)		
(Min-Max: 1–20)		
≤ 10	127	79.9
> 10	32	20.1
Receive training on PPE usage and hand hygiene		
No	53	15
Yes	301	85

*Abbreviations*: $\overline{x}$ mean, *SD* standard deviation

### Perception of COVID-19 infection risk

**Fig.1:**
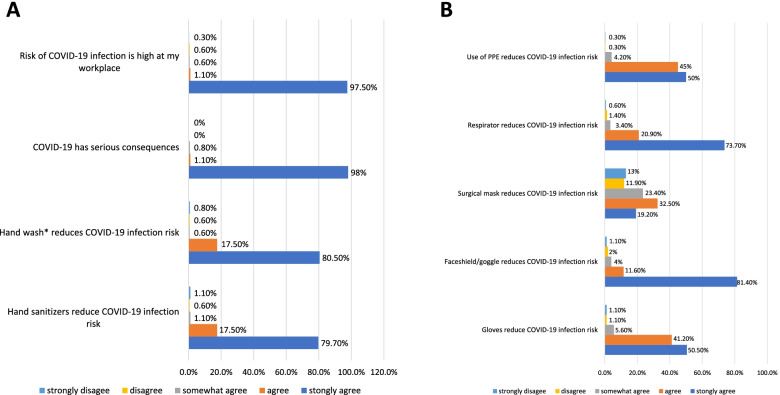
**A** Nurses’ perception of COVID-19 infection risk and risk minimization by hand hygiene, at Alexandria Main University Hospital, 2021 (*n*=354). *With soap and water. **B** Nurses’ perception of COVID-19 infection risk minimization by PPE usage, at Alexandria Main University Hospital, 2021 (*n*=354). Abbreviations: *PPE*, personal protective equipment

Cronbach alpha reliability of the generated scale was 0.68. The mean risk perception score was 40.9 ± 3.3 with a minimum score of 19 and a maximum of 45. More than 95% of nurses believed the high-risk of COVID-19 infection at their workplace, serious consequences of the disease, and minimizing the risk by PPE usage whereas a smaller percentage of nurses strongly believed that the risk of COVID-19 infection could be reduced by the use of surgical mask (19.2%) or gloves (50.5%) (Fig. 1).

### Compliance with PPE usage, hand hygiene, and specific IPC measures

Overall compliance with PPE usage and IPC measures in the nurses was 81.9%. Most nurses showed better compliance with wearing a surgical mask (90.7%) and gloves (88.1%), while less than 10% reported wearing a face shield, goggles, and respirators (FFP3/2, N95), or level 3–4 medical gowns. Most nurses were compliant with the extended use of face masks (99.2%) and a single-use of gloves (98%). More than two thirds of nurses correctly put on and take off PPE for routine care (63.8%) or AGP (76.8%). On the other hand, 43.5% of nurses were not compliant with the recommended steps and duration for hand washing. Similarly, they responded to their activities such as not coming to work for the presence of fever or symptoms (97.7%), maintaining the physical distancing 6 ft. even in non-patient areas (72.3%), and postponing elective time-off during the COVID-19 pandemic (73.7%) (Table 2).

**Table 2:** Compliance with the PPE usage, hand hygiene, and IPC measures among nurses at Alexandria Main University Hospital, 2021 (*n*-354)

	Response	No.	%
**Compliance with PPE usage**			
Wear PPE at work	All times at work^a^	212	59.9
	Only when contact with patient	142	40.1
Wear respirators (FFP3/2, N95)1.	Always, often	5.0	1.4
	Never, rarely, sometimes	349	98.6
Wear surgical mask 1.	Always, often	321	90.7
	Never, rarely, sometimes	33	9.3
Wear Face shield 1.	Always, often	5.0	1.4
	Never, rarely, sometimes	349	98.6
Wear goggles	Always, often	5.0	1.4
	Never, rarely, sometimes	349	98.6
Wear medical gown (level 3 or 4)1.	Always, often	34	9.6
	Never, rarely, sometimes	349	90.4
Wear gloves 1.	Always, often	312	88.1
	Never, rarely, sometimes	42	11.9
**Extended PPE usage**			
Face mask	No	3	0.8
	Yes, per session^b^	351	99.2
Gloves	No	347	98
	Yes, per session^b^	7	2.0
**Donning and doffing PPE**			
Steps for donning PPE	Compliant	226	63.8
	Not compliant	128	36.2
Steps for doffing PPE	Compliant	272	76.8
	Not compliant	82	23.2
**Hand wash with soap and water**			
Steps and duration of hand wash	Compliant	200	56.5
	Not compliant	154	43.5
**Specific IPC measures**			
Not to report to work when having fever, or other symptoms	No	8.0	2.3
	Always, most of times	346	97.7
Maintain physical distancing (6-feet) at work even in non-patient care areas.	No	98	27.7
	Always, most of time	256	72.3
Postpone elective time-off	No	93	26.3
	Always, most of time	261	73.7
**Overall compliance**			
	Good compliance	290	81.9
	Poor compliance	64	18.1

*Abbreviations*: *PPE* Personal protective equipment, *IPC* Infection prevention and control; ^a^Even in non-patient areas, ^b^A single session is a period of time where a healthcare worker is undertaking duties in a specific clinical care setting or exposure environment, for example during a ward round

Regarding the availability of PPE at AMUH, most nurses reported the availability, either always or often, of surgical masks (98.3%), gloves (89.6%), soaps (98.6%), and hand sanitizers (94.6%), whereas face shields/googles (83.9%), and respirators (92.6%) had rarely/never been provided by the hospital. Thirty-five percent of nurses reported that level 3–4 medical gowns were sometimes available. Most nurses (96%) stated that they did not have to buy their PPE.

### Factors associated with compliance with PPE usage, hand hygiene, and IPC measures

Univariate analysis showed that good compliance was significantly associated with a higher level of education (35.9%) and a higher mean perception score (41.3 ± 2.9) compared with the nurses with poor compliance (20.3%; *p* = 0.019, and 39.35 ± 4.7; *p* = <0.001, respectively). The mean age and employment duration were significantly lower among nurses with good compliance (37.6 ± 10.6, and 17.6 ± 11.1, respectively) compared with nurses with poor compliance (41.9 ± 11.1; *p* = 0.005, and 21.5 ± 11.6; *p* = 0.013, respectively) (Table 3).

**Table 3:** Univariate logistic regression analysis of potential factors associated with compliance with PPE, hand hygiene, and IPC measures among nurses at Alexandria Main University Hospital, 2021 (*n*-354)

	Poor compliance (*n*= 64)		Good compliance (*n*=290)		OR (95% CI)	*p* value
	No.	%	No.	%		
Gender						
Man	2	3.5	32	11.8	3.6 (0.8, 15.8)	0.081
Woman^^^	55	96.5	240	88.2		
Level of education						
Nursing school^^^	51	79.7	186	64.1		
Nursing Institute/University	13	20.3	104	35.9	2.2 (1.1, 4.2)	0.019*
History of COVID-19 disease						
No	36	56.3	171	59.0	1.1 (0.6, 1.9)	0.690
Yes^^^	28	43.8	119	41.0		
Colleague/relative had COVID-19 disease						
No	1	1.6	2	0.7	2.0 (0.1, 35.8)	0.638
Yes, recovered	60	93.8	285	98.3	4.7 (0.9, 24.1)	0.060
Yes, died^^^	3	4.7	3	1.0	Ref	-
Department						
Internal medicine	23	35.9	110	37.9	1.1 (0.6, 1.9)	0.766
Surgical & emergency^^^	41	64.1	180	62.1		
Working hours per day (hours)						
≤ 8	52	81.3	220	75.9	0.7 (0.4, 1.4)	0.357
> 8^^^	12	18.8	70	24.1		
Work schedule						
Day time	41	64.1	154	53.1	0.6 (0.4, 1.1)	0.112
Shift work^^^	23	35.9	136	46.9		
Training on PPE usage & hand hygiene						
No	11	17.2	42	14.5	0.8 (0.4, 1.8)	0.759
Yes^^^	53	82.8	248	85.5		
Knowledge						
Min-Max	4–8		4–8			
$\kern1em \overline{x}$ ± SD	5.3 ± 1.04		6.5 ± 0.9		2.8 (2.1, 3.7)	<0.001^***^
Perception						
Min-Max	19–45		28–45			
$\kern1em \overline{x}$ ± SD	39.35 ± 4.7		41.3 ± 2.9		1.1 (1.07, 1.2)	<0.001^***^
Age (years)						
Min-Max	20–59		20–59			
$\kern1em \overline{x}$ ± SD	41.9 ± 11.1		37.6 ± 10.6		0.96 (0.9, 0.98)	0.005^***^
Employment duration (years)						
Min-Max	1.5–40		1.5–40			
$\kern1em \overline{x}$ ± SD	21.5 ± 11.6		17.6 ± 11.1		0.97 (0.9, 0.99)	0.013^**^

*Abbreviations*: *PPE* Personal protective equipment, *IPC* infection prevention and control, $\overline{x}$, mean; *SD* standard deviation, *CI* Confidence interval

^^^reference category

*p≤0.05; **p>0.01; ***p>0.001

Multivariate regression analysis revealed that good compliance with PPE usage and IPC measures was independently predicted by risk perception (OR = 1.25; 95%CI = 1.13, 1.39), and knowledge about PPE usage and hand hygiene (OR =3.53; 95%CI = 2.40, 5.19); those factors were adjusted for other sociodemographic, personal, and occupational factors in the model. The model was able to correctly classify 83.6% of nurses for their compliance (Table 4).

**Table 4:** Multivariate logistic regression analysis of independent predictors of good compliance with PPE, hand hygiene, and IPC measures among nurses at Alexandria Main University Hospital, 2021 (*n*=354)

Independent predictors	Coefficient	Adjusted OR^a^	95% CI	*P* value
Woman gender	1.371	3.938	(0.72, 21.5)	0.114
Higher educational level	0.166	1.181	(0.45, 3.04)	0.731
History of COVID-19 disease	-0.030	0.970	(0.46, 2.03)	0.937
Colleague or relative had COVID-19 disease	-0.890	0.411	(0.02, 7.19)	0.542
Working at surgical and emergency departments	0.101	1.107	(0.54, 2.26)	0.781
Working > 8 h per day	0.519	1.680	(0.55, 5.04)	0.355
Shift work	0.267	1.306	(0.47, 3.59)	0.605
Receiving training on PPE usage & hand hygiene	0.048	1.050	(0.38, 2.88)	0.925
Age	-0.003	0.997	(0.94, 1.09)	0.889
Knowledge	1.262	3.532	(2.40, 5.19)	<0.001^***^
Perception	0.229	1.257	(1.13, 1.39)	<0.001^***^

Model *X*^2^ =82.48 (*p*<0.001); Nagelkerke’s *R*^2^=0.368; Cox & Snell *R*^2^= 0.222; Hosmer & Lemeshow *X*^2^=6.96 (*p*=0.541)

*Abbreviations*: *PPE* Personal protective equipment, *IPC* Infection prevention and control, *OR* Odds ratio, *CI* Confidence interval. ***p<0.001

^a^OR adjusted for all variables in the above table

### Facilitators of compliance with PPE usage as perceived by nurses

**Fig. 2:**
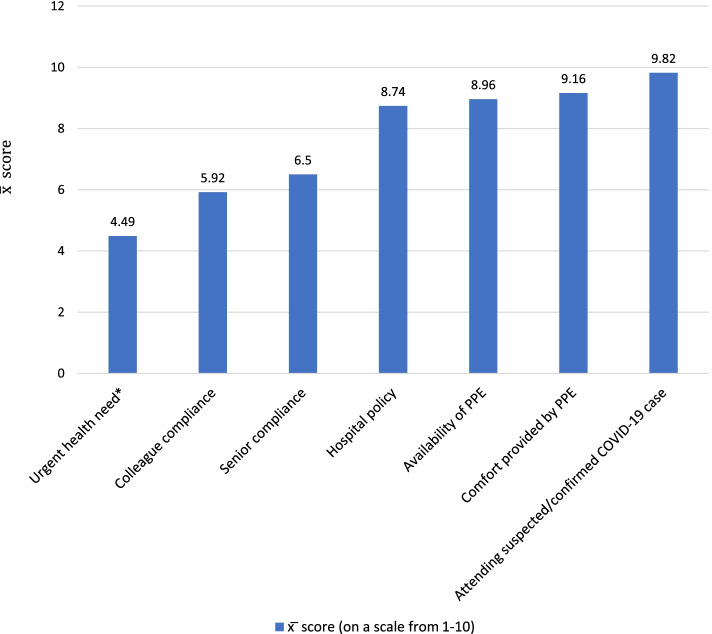
Facilitators of compliance with the PPE usage as perceived by nurses at Alexandria Main University Hospital, 2021 (*n*=354). Abbreviations: $\overline{x}$, mean; *Time allowed before attending a patient; PPE personal protective equipment

Among the reported factors that enhance compliance with PPE usage, the highest mean scores were for attending suspected or confirmed COVID-19 cases ($\overline{x}$ = 9.82), comfort to use PPE ($\overline{x}$ = 9.16), availability of PPE ($\overline{x}$=8.96), hospital policy ($\overline{x}$ = 8.74), and senior compliance ($\overline{x}$ = 6.5) (Fig. 2).

## Discussion

This study revealed the overall compliance rate of the nurses was 81.9%; it was higher than the compliance rate reported in Egypt during the first wave of the pandemic (46.8%) [19], Ethiopia (22%) [17], and Congo (31.5%) [18]. On the other hand, it was consistent with compliance rates reported in studies conducted in Uganda (74%) [14], China (89%) [15], and Pakistan (73%) [16].

The discrepancy in compliance rates reported in different studies might be attributed to the time factor. Some studies were conducted during the first wave of the COVID-19 pandemic [17, 19] when resources at healthcare facilities in many countries were insufficient, perception and knowledge of HCWs were inadequate, and recommendations and guidelines were sometimes inconsistent because the mode of transmission of the virus was not clearly understood. For example, initially, some HCWs believed that PPE is required only when they contact a confirmed COVID-19 case; this had a negative influence on their practice [19, 24]. On the other hand, this study was conducted during the third wave, where the aforementioned factors were all improved.

In Egypt, the steady increase in the number of confirmed COVID-19 cases had not started until June 2020 [19]. Among eleven hospitals affiliated with the Alexandria University, one hospital (Students University Hospital) was dedicated to the management and isolation of moderate to severe confirmed COVID-19 cases. The nurses at Alexandria University hospitals were obligated to rotate to join the health team at the isolation hospital at some point in time. AMUH local IPC teams provided wide training on IPC measures to prepare nurses regarding risky confronts and recommended precautions while providing routine care at Alexandria University hospitals or specific care at the isolation hospital.

Moreover, based on the international guidelines [8–11], the Ministry of Health and Population in Egypt distributed numerous circulars on hospital preparedness and IPC measures [19]. All these factors would explain the high perception, good level of knowledge, and high compliance rate reported among nurses in this study. Similar findings were reported in Abdel Wahed et al. study conducted among HCWs in Egypt [25].

Variation in compliance rates reported in studies could also be explained by the disparity in the studies’ methodology. Self-reporting might overestimate the real compliance rate unlike assessing an observed practice [19]; however, nurses in this study had the opportunity to freely describe their performance as the questionnaire was anonymous. Moreover, variation in the number of items, the measurement scale used to evaluate the compliance, and the inclusion of other professions (such as physicians and technicians) could lead to inconsistency in the findings [24–26].

This study revealed that good compliance with PPE usage, hand hygiene, and IPC measures was independently predicted by nurses’ risk perception and knowledge about PPE usage and hand hygiene. Likewise, Brooks et al. review (2020) studied 56 papers and revealed evidence that staff with higher concern about the risk of infection were more likely to comply with the recommended measures [27]. Similarly, Webster et al. review found that accurate knowledge about the recommended performances, perception of susceptibility and severity of being infected, and perception of benefits of compliance would facilitate compliance [28].

Moreover, in correspondence with facilitators of compliance perceived by nurses in this study, contact with confirmed COVID-19 cases improves HCWs’ compliance, whereas barriers to compliance include PPE unavailability, perceived PPE discomfort, and non-compliance of colleagues at work [27].

### Limitations of the study

Assessment of nurses’ compliance in this study was subjective; it relied on self-rating and description of the practice. Moreover, it would be better to include other professions, to assess the compliance of different professions employed in the same work circumstances.

## Conclusions

At AMUH, during the third wave of COVID-19 in Egypt, nurses reported a high risk perception and rate of compliance with PPE usage, hand hygiene, and IPC measures of 81.9%. The findings indicate that personal perception of COVID-19 infection risk and knowledge about PPE usage and hand hygiene are the keys to improving compliance. Continuous training is recommended to raise nurses’ awareness, knowledge, and perception to ensure good compliance with PPE usage and hand hygiene.

## Data Availability

Data and materials are available. Confidentiality and security of data and materials were ensured through all stages of the study.

## Abbreviations

AGP: Aerosol-generating procedure
AMUH: Alexandria Main University Hospital
CDC: Centers for Disease Control and Prevention
COVID-19: Coronavirus disease of 2019
HCW: Healthcare worker
IPC: Infection prevention and control
PPE: Personal protective equipment
SARS-CoV-2: Severe acute respiratory syndrome coronavirus 2
WHO: World Health Organization
